# Burden of Lung Cancer Attributable to Occupational Carcinogens from 1990 to 2019 and Projections until 2044 in China

**DOI:** 10.3390/cancers14163883

**Published:** 2022-08-11

**Authors:** Yaguang Fan, Yong Jiang, Xin Li, Xuebing Li, Yang Li, Heng Wu, Hongli Pan, Ying Wang, Zhaowei Meng, Qinghua Zhou, Youlin Qiao

**Affiliations:** 1 Tianjin Key Laboratory of Lung Cancer Metastasis and Tumor Microenvironment, Tianjin Lung Cancer Institute, Tianjin Medical University General Hospital, Tianjin 300052, China; 2 National Cancer Center/National Clinical Research Center for Cancer/Cancer Hospital, Chinese Academy of Medical Sciences and Peking Union Medical College, Beijing 100021, China; 3 Department of Lung Cancer Surgery, Tianjin Medical University General Hospital, Tianjin 300052, China; 4 Department of Radiology, Tianjin Medical University General Hospital, Tianjin 300052, China; 5 Department of Nuclear Medicine, Tianjin Medical University General Hospital, Tianjin 300052, China; 6 Sichuan Lung Cancer Institute, Sichuan Lung Cancer Center, West China Hospital, Sichuan University, Chengdu 610041, China; 7 Center of Global Health, School of Population Medicine and Public Health, Chinese Academy of Medical Sciences and Peking Union Medical College, Beijing 100005, China

**Keywords:** lung cancer, disease burden, occupational carcinogen, age-period cohort analysis, decomposition analysis

## Abstract

**Simple Summary:**

The disease burden trend of lung cancer that is attributable to occupational carcinogens in China remains unclear. We used the data from the Global Burden of Disease (GBD) study in 2019 to investigate the related disease burden from 1990 to 2019 and to project the disease burden for the next 25 years. The results indicate that the disease burden of lung cancer that can be attributed to occupational carcinogens significantly increased from 1990 to 2019 in China, and the absolute burden will continue to increase in the next 25 years.

**Abstract:**

Background: Little is known about trends in the lung cancer burden from the disease that can be attributed to occupational carcinogens in China. Methods: Data regarding the lung cancer burden that can be attributed to occupational carcinogens in China were extracted from the Global Burden of Disease (GBD) study in 2019. Joinpoint regression analysis and an age-period-cohort (APC) analysis were conducted to estimate the trend of lung cancer burden as a result of occupational carcinogens from 1990 to 2019. A Bayesian APC model was used to predict the disease burden until 2044. Results: The average annual percentage changes of age-standardized summary exposure values (SEVs) of occupational lung carcinogens, as well as the age-standardized population attributable fraction (PAF) of lung cancer due to occupational carcinogens, were 0.5% (95% confidence interval (CI): 0.4–0.5%) and 0.1% (95% CI: 0–0.2%), respectively. In addition, both the joinpoint regression analysis and APC analysis demonstrated significantly increased trends of age-standardized lung cancer mortality (ASMR) and age-standardized disability-adjusted life years (ASDR) as a result of occupational carcinogens. Asbestos and silica accounted for the two most important occupational lung carcinogens in China. The absolute burden is expected to increase, mainly due to population aging and the age-specific rate of illness. Conclusions: The lung cancer burden that could be attributed to occupational carcinogens significantly increased from 1990 to 2019 in China, and the absolute burden will continue to increase in the next 25 years.

## 1. Introduction

Lung cancer is the most common cancer and is the leading cause of cancer deaths globally, with an estimated 2.1 million new lung cancer cases and 1.8 million deaths in 2018 [1]. In China, lung cancer has been the leading cause of cancer deaths since the 1990s, and the disease burden is set to increase [2]. Cigarette smoking is considered the leading cause of lung cancer [3]. However, many other risk factors, including air pollution, passive smoking, low fruit intake, genetic susceptibility, and occupational exposure to carcinogens have been reported [4,5,6].

Lung cancer is the most common occupation-related cancer, which accounts for 89.0% of attributable cancer deaths [7]. Among the 47 kinds of known specific occupational carcinogens that are classified by the International Agency for Research on Cancer, 19 were associated with the development of lung cancer [8]. However, the disease burden of lung cancer attributed to occupational exposure is controversial, due to the variations in the analytic method, the types of occupational exposure, exposure prevalence, the intensity of exposure, and the regional/ethical background [9,10,11,12].

China is a rapidly developing country that has experienced marked socio-economic changes. However, this has led to a high disease burden that is attributable to occupational risks [13]. In our previous report, we estimated that in 2005, 30,387 men and 9471 women died from lung cancer that was attributable to occupational exposure in China [3]. However, little is known about the trends in recent decades in China regarding the disease burden of lung cancer that is attributable to occupational exposure. Furthermore, the burden of lung cancer caused by occupational carcinogens may be influenced by educational level, socioeconomic status, and the synergistic effect with other carcinogens. These risk factors might vary with chronological age, time period, and birth cohort. Traditional descriptive analysis cannot eliminate these confounding effects. Conversely, age-period cohort models offer substantial advantages when evaluating the age, time period, and cohort effects on the disease burden [14,15].

The GBD study estimates a variety of metrics of diseases recorded annually, dating back to 1990. This provides a unique opportunity to assess the long-term trends of the disease burden in relation to various risk factors. Based on the GBD data, several studies investigated the disease burden attributable to occupational risks for cancer in China or worldwide [7,13]. However, little is known regarding the time trend and the current status of the disease burden of occupational lung cancer in China. In this study, we analyzed the trend of the lung cancer burden attributable to occupational carcinogens from 1990 to 2019, then projected the disease burden for the next 25 years and decomposed the changes in the predicted number of lung cancer deaths and disability-adjusted life-years (DALYs) in terms of population growth, population aging, and epidemiological changes, in order to provide useful information for occupational lung cancer control in China.

## 2. Materials and Methods

### 2.1. Data Source

Data regarding the disease burden of lung cancer that can be attributed to occupational carcinogens in China were derived from the GBD 2019 [16]. The GBD 2019 followed the general framework established for comparative risk assessment and provided annual disease burden estimates of 369 diseases and injuries for 87 risk factors and combinations of risk factors, in 204 countries and territories, from 1990 to 2019. The detailed methodology in the GBD 2019 estimation process, including the risk factor hierarchy, determining the inclusion of risk–outcome pairs, estimating relative risk as a function of exposure for each risk–outcome pair, estimating the distribution of exposure for each risk, estimating the population attributable fraction and attributable burden according to age-sex-location-year, and establishing the summary exposure value has been fully described elsewhere [16]. Data in China were obtained from the China Center for Disease Control and Prevention and the Maternal and Child Health Surveillance System [17]. We chose “China” from the database as the location, “tracheal, bronchial, and lung cancer” as the cause, “Occupational carcinogen” as the risk, and “death” and “DALYs” as the measures. Data were downloaded from the Global Health Data Exchange website (https://ghdx.healthdata.org/gbd-results-tool, accessed date: 20 April 2022). As a secondary analysis of publicly available data, no ethical approval from an institutional review board was required for this study.

### 2.2. Occupational Carcinogens Associated with Lung Cancer

A total of 13 work-environment carcinogens associated with 7 cancer types were assessed in GBD 2019. In this study, we focused on the disease burden of lung cancer that can be attributed to 9 occupational carcinogens, including arsenic, asbestos, beryllium, cadmium, chromium, diesel engine exhaust, nickel, polycyclic aromatic hydrocarbons, and silica. The individual and combined age-standardized SEVs for occupational carcinogens that are associated with lung cancer were obtained. A SEV was developed for GBD 2015, in terms of the relative risk-weighted prevalence of exposure. The SEV ranged from zero, when no excess risk exists in a population, to one, when the population is at the greatest risk.

### 2.3. Disease Burden Metrics

Data on the annual number of lung cancer-related deaths, DALYs, ASMR, ASDR, population attributable fraction (PAF), and age-standardized PAF that were attributable to occupational carcinogens from 1990 to 2019 were obtained. Attributable deaths and DALYs were estimated using the total resulting death rate or DALYs, multiplied by the PAF for the risk-outcome pair for each age, sex, cause, and location. The detailed computation process of PAF was described in a previous study [7]. The age standardization that we used was based on the GBD 2019 global population figures.

### 2.4. Uncertainty Estimation Interval

The uncertainty estimation interval (UI) was calculated to address the possible heterogeneity issue from both the sampling error and non-sampling variance for GBD 2019. The 95% UI was calculated by taking 1000 samples from the posterior distribution of the respective step in the modeling process and was reported as the 2.5th and 97.5th values for each estimate. The UIs for SEV and the number/rate/PAF of lung cancer incidences attributable to occupational carcinogens were also obtained directly from the Global Health Data Exchange website.

### 2.5. Statistical Analysis

Descriptive analyses were conducted on lung cancer cases that were attributable to occupational carcinogens between the years of 1990 and 1999 in China. The percentage change was calculated to describe the trend of the measures. The trends of age-standardized SEV, ASMR, ASDR, and age-standardized PAF were further assessed using joinpoint regression analysis on a log scale, since these measures followed a Poisson distribution [18]. Joinpoint regression analysis uses piecewise linear regression to estimate the adaptive trend using one or more line segments. The analysis starts with the minimum number of joinpoints (i.e., the 0 joinpoint, which is a straight line) and tests whether one or more joinpoints are statistically significant and must be added to the model, using a Monte Carlo permutation method. An annual percentage change in ASMRs and ASDRs for each line segment was estimated. The average annual percentage change and the related 95% CI were also computed to represent a summary measure of the trend during the full study period from 1990 to 2019. Z tests were used to assess whether the annual percentage changes and average annual percentage changes were significantly different from zero.

Furthermore, we used an APC model to analyze the effects of age, period, and cohort on mortality and the DALYs of lung cancer cases attributable to occupational carcinogens. The APC model was based on a log-linear model for the rate, with additive effects from age, calendar period, and birth cohort, as shown in the formula below [19]:

Logλ_ij_ = μ + α_i_ + β_j_ + γ_k_ i = 1, 2, …, I,

           j = 1, 2, …, J,

          k = j − i + I.

In the above model, μ refers to the intercept term, α_i_ refers to the age effects, β_j_ refers to the period effects, and γ_k_ refers to the cohort.

We performed the APC analysis with a freely available web tool (http://analysistools.nci.nih.gov/apc/, accessed date: 20 April 2022) [20]. The APC functions provided by this web tool included: (1) net drift, which represents the annual percentage change of the expected age-adjusted rates over time, (2) local drifts, which assess the annual percentage change of the expected age-specific rates over time, (3) the longitudinal age curve, which shows the fitted longitudinal age-specific rates in the reference cohort, adjusted for period deviations, (4) period rate ratios (RRs), the ratio of age-specific rates in each period relative to the reference period, and (5) cohort RRs, the ratio of age-specific rates in each cohort relative to the reference cohort. The longitudinal age curve, time period RR, and cohort RR represent the age, period, and cohort effect, respectively. The central age group, period, and birth cohort were defined as the references in the APC analysis, respectively.

We predicted the number of deaths, DALYs, ASMR, and ASDR of occupational carcinogen-attributable lung cancer from 2019 to 2044, with a Bayesian APC model of integrated nested Laplace approximations (the R packages, BAPC and INLA), assuming the inverse gamma prior distribution of age, period, and cohort effects and applying a second-order random walk (RW2) to adjust for excessive dispersion [21]. The estimated population of China from 2020 to 2044 was obtained from the 2019 revision of the United Nations (UN) World Population Prospects, by year (up to 2100), age, and sex.

In addition, we used a newly developed decomposition method to attribute changes in the total number of lung cancer deaths and DALYs, from occupational carcinogens to population growth, population aging, and age-specific changes between 1990 and each subsequent year from 1991 to 2044 [22,23]. The absolute and relative contributions of the three drivers to the change in the number of lung cancer deaths and DALYs attributable to occupational carcinogens were calculated, and the numbers of 1990 were taken as references. A positive contribution indicates an increase in total deaths/DALYs. The age-specific changes represent the epidemiological profile of occupational lung carcinogens that cannot be explained by population growth and population aging. The net changes in these three drivers are equal to the difference in the total number of observed deaths and DALYs. Statistical significance was defined as a two-sided *p*-value of less than 0.05.

## 3. Results

### 3.1. Trends in the Lung Cancer Burden Attributable to Occupational Carcinogens

In 2019, among 67,264 deaths and 1,639,492 DALYs from occupational carcinogen-attributable cancer, 93.5% (62,861) deaths and 92.1% (1,509,863) DALYs were from lung cancer. The estimated number, all-age and age-standardized PAF, mortality, and the DALYs of lung cancer due to occupational carcinogens in 1990 and 2019 are presented in Table 1. Although the percentage changes for the number of deaths and DALYs increased significantly from 1990 to 2019, the significant increase in percentage change was only observed for death rates in terms of ASMR (0.31, 95% UI: 0.01–0.71), not for ASDR (0.14, 95% UI: −0.12–0.50). Besides this, no significant increases were observed for all-age PAF and age-standardized PAF, in terms of the percentage change. As also shown in Table 1, the age-standardized SEVs of occupational lung carcinogens collectively increased significantly from 1990 to 1999, with a percentage change of 0.14 (95% UI: 0.07–0.23).

**Figure 1:**
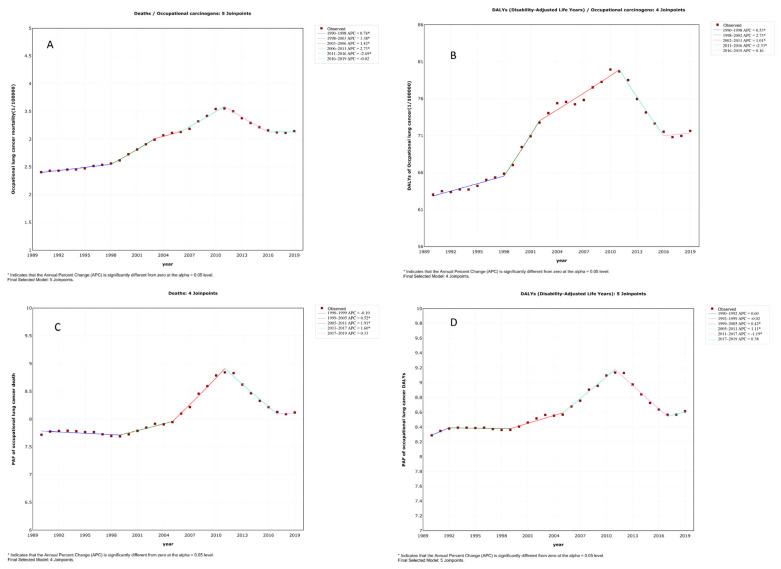
Joinpoint regression analysis of ASMR, ASDR, age-standardized PAF of death rates and DALYs due to lung cancer that could be attributed to occupational carcinogens, recorded from 1990 to 2019, described by the slope of the graph as the annual percentage change for each time segment. (**A**) ASMR with 6 time segments (5 joinpoints). (**B**) ASDR with 5 time segments (4 joinpoints). (**C**) Age-standardized PAF of lung cancer deaths that can be attributed to occupational carcinogens with 5 time segments (4 joinpoints). (**D**) Age-standardized PAF of lung cancer DALYs that can be attributed to occupational carcinogens with 6 time segments (5 joinpoints).

According to the results of the joinpoint regression model, the average annual percentage change of the age-standardized SEV of occupational carcinogens, taken collectively, was 0.5% (95% CI: 0.4–0.5%). Furthermore, the average annual percentage changes for ASMR, ASDR, the age-standardized PAF for death rates and age-standardized PAF for DALYs were 0.9% (95% CI: 0.7–1.1%), 0.4% (95% CI: 0.2–0.7%), 0.1% (95% CI: 0–0.3%), and 0.1% (95% CI: 0–0.2%), respectively. All of them demonstrated a significantly increased trend. However, these trends fluctuated in different segments. As shown in Figure 1, the age-standardized rates of both occupational lung cancer death rates and DALYs were all significantly increased from 1990 to 2011 and were more predominant from 1998 to 2002. Subsequently, significant decreasing trends were observed. Similar patterns were observed for age-standardized PAFs of both death rates and DALYs of lung cancer cases that were attributable to occupational carcinogens. In contrast, the age-standardized SEV of occupational carcinogens increased significantly in the last three years of the study period (Supplemental Figure S1).

The trends of age-standardized SEVs, PAF and ASMR, and the ASDR of lung cancer cases that were attributed to individual occupational carcinogens are shown in Supplemental Tables S1–S3. Silica and asbestos were the two most important occupational carcinogens that have been associated withed lung cancer, followed by diesel engine exhaust. From 1990 to 2019, asbestos replaced silica as the most important carcinogen in terms of age-standardized PAF and ASMR in terms of recorded deaths, while silica still accounted for the highest attribution fraction of occupational lung cancers in terms of age-standardized PAF and ASDR for DALYs.

### 3.2. APC Analysis of the Lung Cancer Burden Attributable to Occupational Carcinogens

**Figure 2:**
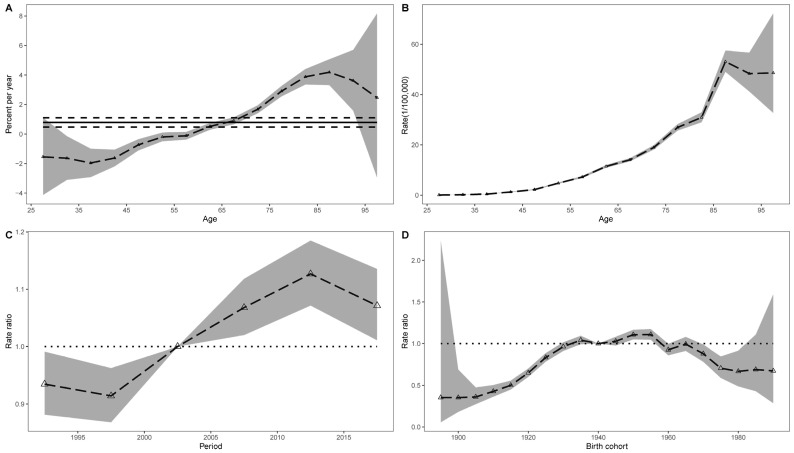
Effects of age, time period, and cohort on lung cancer deaths attributed to occupational carcinogens from 1990 to 2019 in China. (**A**) Local drifts with net drift values; the horizontal solid and dashed lines represent the net drift and its 95% CI respectively, the dashed line and shaded area represent the local drifts and their 95% CIs. (**B**), Fitted longitudinal age curves of mortality of lung cancer, the dashed line and shaded area represent age-specific lung cancer mortality due to occupational carcinogens and their 95% CIs, respectively. (**C**) Relative risks of each period compared with the reference period (2000–2004) (dashed line with triangle), adjusted for age and cohort effects, and the corresponding 95% CIs (shaded area). (**D**) Relative risks of each cohort compared with the reference cohort (cohort 1940–1944) (dashed line with triangle), adjusted for age and time period effects, and the corresponding 95% CIs (shaded area).

Figure 2 demonstrated the effects of age, time period, and cohort on deaths from lung cancers that could be attributed to occupational carcinogens, as recorded in age groups from 25 to 95-plus from 1990 to 2019. The overall annual percentage change (net drift) across the study period was 0.79 (95% CI: 0.48–1.10) (Figure 2A). This suggested a significantly increased trend of lung cancer deaths that were attributable to occupational carcinogens, after excluding the effects of age and birth cohort. Moreover, this trend varied significantly according to age (local drifts), and the percentages were below 0 in those subjects under the age of 60, which indicated a decreased trend in these age groups. The longitudinal age curve of occupational cancer mortality is illustrated in Figure 2B. The risk of occupational cancer increased monotonically except for minor decreases at advanced ages (85 years and above), which might be explained by competing risks from other causes of death.

**Figure 3:**
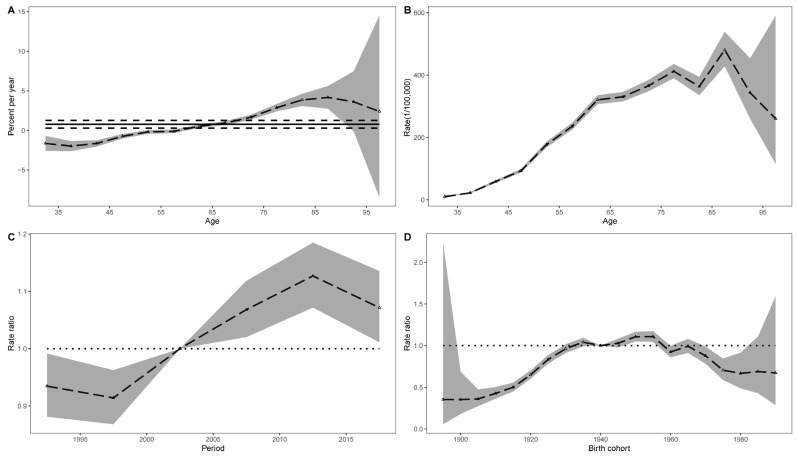
Effects of age, time period, and cohort on lung cancer DALYs attributable to occupational carcinogens from 1990 to 2019 in China. (**A**) Local drifts with net drift values, the horizontal solid and dashed lines represent the net drift and its 95% CI respectively, the dashed line and shaded area represent the local drifts and their 95% CIs. (**B**) Fitted longitudinal age curves of DALYs rate of lung cancer, the dashed line and shaded area represent age-specific rate of lung cancer DALYs due to occupational carcinogens and their 95% CIs, respectively. (**C**) Relative risks of each period compared with the reference period (2000–2004) adjusted for age and cohort effects (dashed line with triangle) and the corresponding 95% CIs (shaded area). (**D**) Relative risks of each cohort compared with the reference cohort (cohort 1940–1944) (dashed line with triangle), adjusted for age and time period effects and the corresponding 95% CIs (shaded area).

The estimated period and cohort RRs for lung cancer deaths attributable to occupational carcinogens are displayed in Figure 2C,D. The time-period RRs were gradually increased from 1997 to 2012 and then showed a downward trend. The risk of lung cancer death increased in the first several birth cohorts and declined in cohorts born after 1960. In addition, the results of the Wald tests suggested that the net drifts, local drift, cohort, and time period effects were all statistically significant. Similar patterns were observed for the age, time period, and cohort effects on the DALYs of lung cancer associated with occupational carcinogens (Figure 3A–D).

The effects of age, time period, and cohort on the death rates and DALYs of lung cancer that can be attributed to individual occupational carcinogens are shown in Supplemental Tables S4–S8. A significantly increased disease burden was only observed for asbestos, with net drifts of ASMR and ASDR of 2.39 (95% CI: 0.99, 3.81) and 2.42 (95% CI: 1.56, 3.29), respectively. The net drifts of ASMR and ASDR for silica were insignificant; both the significantly decreased local drifts in those under 60 years old and the lower lung cancer risk in cohorts born after 1960 imply that the disease burden from silica will continue to decline.

### 3.3. Prediction and Decomposition Analysis of Lung Cancer Burden

**Figure 4:**
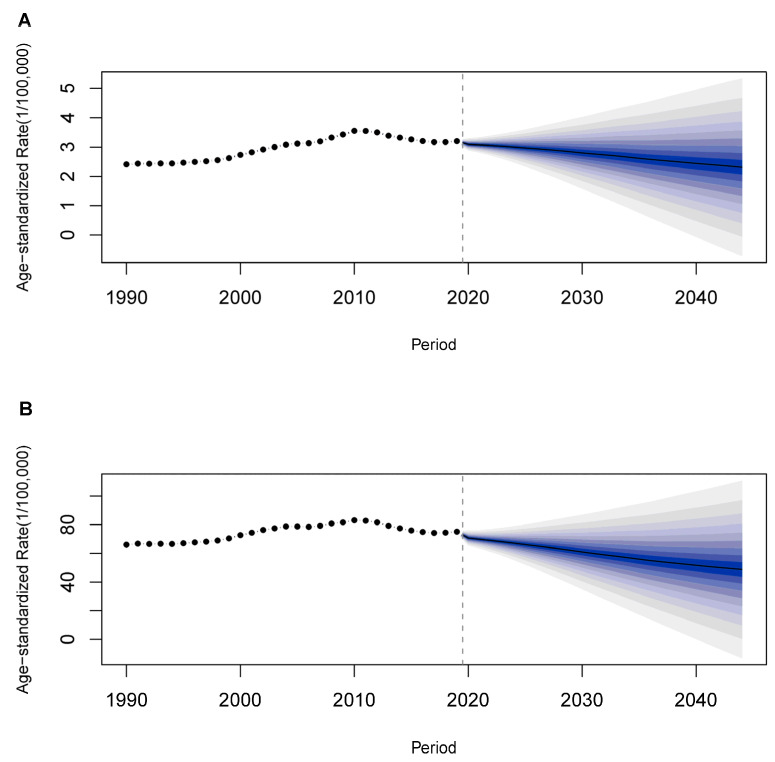
The temporal trends of ASMR and ASDR for lung cancer that can be attributed to occupational carcinogens between 1990 and 2044 in China. The predictive mean is shown as a solid line. The dotted line represents the observational values from the GBD dataset. The vertical dashed line indicates where the prediction starts. The predictive mean value is shown as a black solid line. The fan shows the predictive distribution between the 5% and 95% quintiles, whereby the shaded bands show prediction intervals in increments of 10%. (**A**) ASMR. (**B**) ASDR.

**Figure 5:**
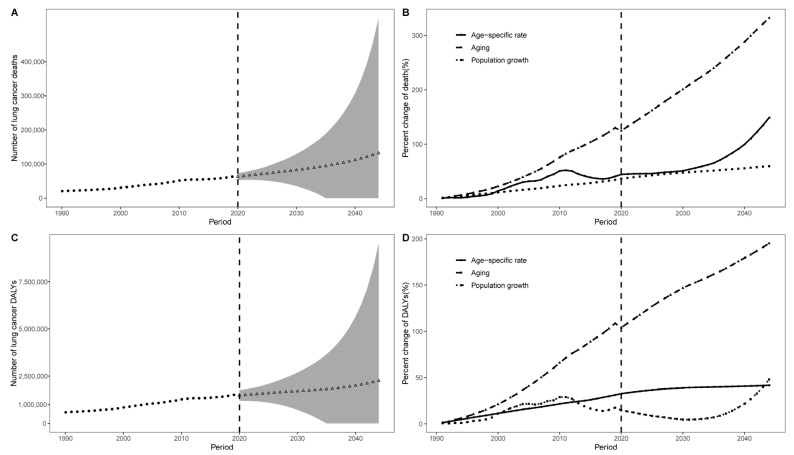
Trends and contribution changes of the number of lung cancer deaths and DALYs that are attributable to occupational carcinogens from 1990 to 1999. (**A**), The trend of the observed (dotted line, 1990–2019) and predicted (triangle line, 2020–2044) numbers of lung cancer deaths. (**B**) Contribution of changes in population aging, population growth, and age-specific rate in the number of lung cancer deaths. (**C**). The trend of observed (dotted line, 1990–2019) and predicted (triangle line, 2020–2044) numbers of lung cancer DALYs. (**D**) Contribution of the changes in population aging, population growth, and age-specific rates in terms of the number of lung cancer DALYs. Data points to the left of the vertical dashed line represent a trend or decomposition based on observed data, while the data points to the right of the line represent the projected data. The shaded areas in (**A**,**C**) show a 95% predictive distribution.

Finally, we conducted a Bayesian APC analysis to project the future trends of the ASMR and ASDR of lung cancer that can be attributable to occupational carcinogens from 2020 to 2044 in China. The age-specific rates of death and DALYs were shown in Supplemental Tables S9 and S10. The ASMR and ASDR would decrease gradually in China (Figure 4). However, the number of occupational lung cancer deaths and DALYs are expected to continue to increase in China during the next 25 years. There were 111,195 additional occupational carcinogen-attributed lung cancer deaths in China in 2044 compared to 1990, an increase of 540.5%. Decomposition analysis result suggests that this increase was due to population aging (332.0%), age-specific rate (148.8%), and population growth (59.7%), respectively. The number of lung cancer DALYs due to occupational carcinogens increased from 586,721 in 1990 to 1,672,111 (285.0%) in 2044, which was driven by population aging (195.2%), age-specific rate (48.0%) and population growth (41.8%), respectively (Figure 5, Supplemental Tables S11 and S12).

## 4. Discussion

In this study, based on data from the GBD 2019, we found that the lung cancer burden that is attributable to occupational carcinogens still increased in terms of ASMR, ASDR, and age-standardized PAF in China. However, these trends fluctuated in different time segments from 1990 to 2019. After adjusting for the age and cohort effects with the APC model, similar trends were also observed for ASMR and ASDR. Although both the ASMR and the ASDR will decline in the coming 25 years, the numbers of both deaths and DALYs will continue to increase.

The significantly increased trend of ASMR and ASDR for lung cancer that can be attributed to occupational carcinogens might mainly result from the high prevalence of occupational exposure, which was a reflection of the rapid economic development and increased industrialization processes in China in previous decades. This finding can be supported by the significant increase in the SEVs of occupational lung carcinogens from 1990 to 2019 seen in our study. A previous study also reported the elevated trend of SEVs of the most common occupational risks in China from 1990 to 2017 [13].

However, the above analysis could not eliminate the confounding effects of age and cohort, and the results may have some limitations [20]. Therefore, we conducted a further APC analysis. We found that the period RRs for occupational carcinogens associated with lung cancer mortality/DALYs had increased since 1990 and reached the highest values during the period of 2010–2015. Moreover, birth cohort effects represent the influence of physical and social exposures that appear earlier in the life process and will accumulate as time progresses [24]. In this study, the cohort RRs for occupational carcinogens that are associated with lung cancer mortality/DALYs showed an increasing trend for those cohorts born before 1950.

Results from the joinpoint analysis demonstrated that from 2011 to 2016, a significant reduction in ASMR and ASDR for lung cancer could be attributed to occupational carcinogens. Similarly, in the APC model, time-period RRs also showed a downward trend since 2012, after adjusting for the confounding effects of age and cohort. Furthermore, we found that the local drift values were lower than 0, while both the period RRs after 2015 and the cohort RRs in those born after 1960 demonstrated decreased trends. These results might be due to the long-term continued efforts regarding the control and prevention of occupational exposure to carcinogens in China, which included policy-making and legislation, occupational supervision, occupational surveillance, labor protection, etc. [25,26]. Furthermore, these findings offered reasonable evidence for the expected drop in ASMR and ASDR of lung cancers that can be attributed to occupational carcinogens during the next 30 years of this study.

More advanced age is associated with lung cancer. In this study, the rates of both deaths and DALYs regarding occupational carcinogens that can be associated with lung cancer are low during their youth and increase with age, reaching a peak among the 85–90-year age group, which might be due to both age-related biological factors and the increased level of occupational exposure. Consequently, the rapid aging process, the rising life expectancy, and longer exposure to occupational carcinogens in China might be the main reasons for the continuing increased number of deaths and DALYs of occupational carcinogen-attributable lung cancer predicted by the Bayesian APC model [27,28,29], which had high coverage [30,31]. Decomposition analysis also confirmed that population aging contributed to most of the additional lung cancer deaths and DALYs that were attributable to occupational carcinogens.

Previous studies found that asbestos was responsible for the largest number of lung cancer deaths and DALYs globally. However, its attributable rates of death and DALYs have decreased significantly since 1990 [7,32]. This was mainly due to the fact that most developed countries have implemented complete bans on the use of asbestos. However, in some developing countries, especially in Asian countries such as China and India, the manufacturers continue to use large quantities of asbestos [33,34]. Accordingly, as shown in this study, the lung cancer burden from asbestos still increased from 1990 to 2019, while asbestos ranked first in terms of deaths from lung cancer that were attributable to occupational carcinogens.

Occupational exposure to silica is very common in certain industries, such as metal or coal mining, construction, painting, glass production, and foundries. Silica exposure has consistently been associated with lung cancer [35]. It is estimated that over 23 million workers in China have been either directly or indirectly exposed to silica [36]. The lung cancer burden that was attributable to silica was second only to that of asbestos [7,37]. Over the last three decades, both the number of deaths and the age-standardized mortality have increased. However, there was a significantly decreased trend of ASMR in regions with high and middle-high social development indexes. In contrast, the ASMR increased significantly in regions with low-middle and low social development indexes [37]. In this study, although the changes in its ASMR and ASDR were insignificant, the SEV, the age-standardized PAF of deaths, and the DALYs from silica showed a significantly decreased trend from 1990 to 2019 in China. In addition, the annual percentage change significantly declined in those less than 60 years old in the APC analysis, which implied that the disease burden of lung cancer that is attributable to silica might not increase in the future.

This is the first study to investigate the impacts of age and time period on cohorts regarding the temporal trends of lung cancer burden caused by occupational carcinogens in China. The predicted increase in the absolute lung cancer burden due to occupational carcinogens emphasizes the importance of improving occupational health in China. Because lung cancer was the most common cancer type affected by occupational carcinogens, a comprehensive strategy integrating significant resources from governments, employers, workers, and other stakeholders to prevent and control occupational lung cancer should be developed. In particular, the prevention and control of asbestos and silicon use should be strengthened.

The global burden of lung cancers due to occupational carcinogens is decreasing [32,37]. However, the trends differed between regions, based on the social development index. Although ASDR is decreasing in countries with high social development indexes, the death toll remains high, owing primarily to population growth and aging. However, similar to China, the lung cancer burden attributable to occupational carcinogens increased significantly from 1990 to 1999 in countries with low, low–middle, and middle social development indexes. Accordingly, a precise estimate of the burden of lung cancer related to occupational carcinogens, along with the consideration of demographic drivers was a critical component for occupational lung cancer control.

Some limitations, as highlighted in earlier GBD study reports, should be addressed [38,39]. Firstly, despite the fact that erionite, chloromethyl methyl ether, and bis (chloromethyl) ether were recognized as lawful occupational carcinogens in China, the disease burden of lung cancer caused by these occupational carcinogens was not estimated [40]. Secondly, the prevalence of occupational exposure was primarily based on the CAREX (carcinogen exposure) database, which was derived from 15 countries in the European Union from 1990 to 1993 [41]. Similarly, the relative risks used in systematic reviews in the GBD study were also derived from prospective observational studies and case-control studies that were conducted in developed countries. Thirdly, the potential interactions between occupational carcinogens and other risk factors were not taken into account. For example, it was reported that there was a significant super-additive interaction between asbestos and smoking for lung cancer risk [42]. Finally, the precision (prediction interval) in terms of longer projection years was less accurate. Subsequently, the predicted burden of lung cancer that can be attributed to occupational carcinogens might be less precise.

## 5. Conclusions

The lung cancer burden due to occupational carcinogens still increased from 1990 to 2019 in China. Although the predicted ASMR and ASDR of lung cancer that was attributable to occupational carcinogens showed a significant declining trend, the absolute number will still increase in the next 25 years due to the rapid population aging seen in China. This study provides guidance for prevention and control programs for occupational lung carcinogens, especially in the case of asbestos and silica.

## Figures and Tables

**Table 1:** The SEV and burden of lung cancer cases that can be attributed to occupational lung carcinogens.

Metric	Measure	Year		Percentage Change (UI)
		1990	1999	
SEV	All ages (%)	1.01 (0.81–1.45)	1.17 (0.94–1.68)	0.16 (0.09–0.25)
	Age-standardized (%)	0.99 (0.81–1.42)	1.14 (0.92–1.67)	0.14 (0.07–0.23)
Death	Numbers (n)	20,572 (14,227–27,751)	62,861 (43,949–84,923)	2.06 (1.36–3.05)
	PAF (all ages, %)	8.03 (5.59–10.74)	8.30 (6.24–10.68)	3.35 (−8.55–19.97)
	Age-standardized PAF (%)	7.72 (5.55–10.12)	8.12 (6.14–10.48)	3.20 (−7.69–20.79)
	Mortality (All ages, 1/105)	1.74 (1.20–2.34)	4.42 (3.09–5.97)	1.54 (0.96–2.37)
	ASMR (1/105)	2.41 (1.69–3.25)	3.14 (2.20–4.24)	0.31 (0.01–0.71)
DALYs	Numbers (n)	586,721 (395,416–807,333)	1,509,864 (1,035,408–2,075,488)	1.57 (0.99–2.41)
	PAF (all ages, %)	8.43 (5.77–11.45)	8.81 (6.43–11.57)	4.49 (−5.73–17.85)
	Age-standardized PAF (%)	8.29 (5.73–11.13)	8.61 (6.32–11.26)	3.94 (−6.77–17.30)
	All-age rate (1/105)	49.57 (33.41–68.21)	106.15 (72.80–145.92)	1.14 (0.65–1.84)
	ASDR (1/105)	63.03 (33.41–85.88)	71.65 (49.31–98.05)	0.14 (−0.12–0.50)

## Data Availability

The datasets generated for this study can be found in the GBD at http://ghdx.healthdata.org/gbd-results-tool (accessed date: 20 April 2022).

## Supplementary Materials

The following supporting information can be downloaded at: https://www.mdpi.com/article/10.3390/cancers14163883/s1, Figure S1: Joinpoint regression analysis of SEVs of occupational lung carcinogens; Table S1: Age-specific summary exposure value for occupational carcinogens in China, 1990–2019; Table S2: Trend of age-standardized PAF of occupational carcinogens for lung cancer death and DALYs in China, 1990–2019; Table S3: Trends of ASMR and ASDR of lung cancer attributable to occupational carcinogens in China, 1990–2019; Table S4: Age-specific annual percentage change(Local drift) of occupational carcinogen attributable lung cancer ASMR in China from 1990 to 2019; Table S5: Age-specific annual percentage change (local drift) of occupational carcinogen attributable lung cancer ASDR in China from 1990 to 2019; Table S6 Period effect of occupational carcinogen attributable lung cancer ASDR and ASMR in China from 1990 to 2019; Table S7: Birth cohort effect of occupational carcinogen attributable lung cancer ASMR in China from 1990 to 2019; Table S8: Birth cohort effect of occupational carcinogens associated with lung cancer ASDR in China from 1990 to 2019 Cohort-dalys; Table S9: Predicted age-specific rate of lung cancer death attributable to occupational carcinogens; Table S10: Predicted age-specific rate of lung cancer DALYs attributable to occupational carcinogens; Table S11: Contribution of changes in population aging, population growth, and age-specific death rate to the net change of lung cancer from occupational carcinogens in China from 1991 to 2044; Table S12: Contribution of changes in population aging, population growth, and age-specific rate to the net change of lung cancer DALYs from occupational carcinogens in China from 1991 to 2044.
