# Supplementary material for: Burden of Lung Cancer Attributable to Occupational Carcinogens from 1990 to 2019 and Projections until 2044 in China
Source: Cancers (Basel). 2022 Aug 11;14(16):3883. doi: 10.3390/cancers14163883 (PMC9405822; doi:10.3390/cancers14163883)
Supplement: Supplementary file 1 [file cancers-14-03883-s001.zip › cancers-1775604-supplementary/cancers-1775604-supplementary table1.pdf]

Table S1 Age-specific summary exposure value for occupational carcinogens in China,1990-2019

Exposure	Year		Percent change	Average annual percentage change
	1990	1999		
Silica	4.85(2.57-10.64)	4.61(2.46-10.04)	-4.93(-11.68-2.61)	-0.2(-0.2,-0.1)*
Diesel engine exhaust	2.21(2.15-2.28)	2.62(2.47-2.77)	18.32(11.00-25.86)	0.6(0.5-0.6)*
Polycyclic aromatic hydrocarbons	1.07(1.04-1.1)	1.25(1.17-1.35)	17.25(8.37-27.41)	0.5(0.5-0.6)*
Asbestos	0.73(0.53-1.08)	0.78(0.61-0.96)	7.81(-31.88-58.85)	0.2(0-0.4)
Nickel	0.56(0.19-1.49)	0.56(0.18-1.45)	0.51(-6.49-8.98)	0(0-0.1)
Chromium	0.53(0.51-0.55)	0.63(0.59-0.68)	18.69(9.4-29.55)	0.6(0.5-0.7)*
Arsenic	0.51(0.21-0.87)	0.57(0.23-0.97)	10.55(2.04-20.98)	0.3(0.3-0.4)*
Cadmium	0.27(0.26-0.28)	0.3(0.28-0.32)	10.71(2.83-19.76)	0.4(0.3-0.4)*
Beryllium	0.14(0.14-0.14)	0.14(0.14-0.14)	0.52(-2.09-3.49)	0(0-0.1)

* Average annual percentage change was significantly different from 0 at the alpha=0.05 level

Table S2 Trend of age-standardized PAF of occupational carcinogens for lung cancer death and DALYs in China,1990-2019

Measure	Exposure	Year 1990	Year 2019	Percent change	Average annual percentage change
Death	Silica	2.86(1.31-4.51)	2.41(1.08-3.73)	-15.81(-22.46,-8.97)	-0.6(-0.7,-0.5)*
	Asbestos	2.46(1.56-3.79)	3.4(2.19-4.85)	38.07(-3.83,84.4)	1.0(0.9,1.2)*
	Diesel engine exhaust	1.05(0.92-1.2)	1.09(0.95-1.24)	3.29(-4.96,12.12)	0.1(0,0.2)*
	Nickel	0.57(0.11-1.26)	0.49(0.1-1.09)	-13.66(-20.2,-6.62)	-0.5(-0.6,-0.4)*
	Arsenic	0.51(0.19-0.83)	0.48(0.18-0.79)	-5.94(-13.71,2.29)	-0.2(-0.3,-0.1)*
	Polycyclic aromatic hydrocarbons	0.33(0.28-0.38)	0.32(0.27-0.38)	-0.66(-8.95,8.63)	0(-0.1,0.1)
	Chromium	0.09(0.08-0.1)	0.09(0.08-0.11)	0.24(-8.25,8.87)	0(-0.1,0.1)
	Cadmium	0.05(0.04-0.06)	0.05(0.04-0.06)	-5.58(-13.61,3.25)	-0.2(-0.3,-0.1)*
	Beryllium	0.02(0.02-0.03)	0.02(0.02-0.02)	-11.32(-17.08,-5.4)	-0.4(-0.5,-0.3)*
DALYs	Silica	3.55(1.62-5.58)	3.11(1.39-4.8)	-12.54(-18.94,-5.89)	-0.5(-0.5,-0.4)*
	Asbestos	1.75(1.08-2.73)	2.51(1.59-3.66)	43.3(-5.03,100.09)	1.2(1.0-1.4)*
	DE	1.31(1.15-1.48)	1.4(1.24-1.6)	7.33(-0.5,15.7)	0.2(0.2-0.3)*
	Nickel	0.7(0.14-1.56)	0.63(0.12-1.41)	-10.1(-16.42,-3.11)	-0.4(-0.4,-0.3)*
	Arsenic	0.63(0.24-1.03)	0.62(0.23-1.02)	-1.9(-9.63,6.07)	-0.1(-0.2,0)
	PAH	0.4(0.34-0.47)	0.42(0.35-0.49)	3.63(-4.76,13.04)	0.1(0-0.2)*
	Chromium	0.12(0.1-0.13)	0.12(0.11-0.14)	4.65(-3.74,13.17)	0.2(0.1,0.2)
	Cadmium	0.06(0.05-0.07)	0.06(0.05-0.07)	-1.53(-9.51,7.12)	-0.1(-0.1,0)
	Beryllium	0.03(0.02-0.03)	0.03(0.02-0.03)	-7.96(-13.01,-2.53)	-0.3(-0.4,-0.2)*

* Average annual percentage change was significantly different from 0 at the alpha=0.05 level

Table S3 Trends of ASMR, ASDR of lung cancer attributable to occupational carcinogens in China,1990-2019

Measure	Carcinogen	Year		Percent change	Average annual percentage change	Netdrift
		1990	1999			
ASMR	Silica	0.89(0.41-1.43)	0.93(0.41-1.54)	4.59(-19.14-33.68)	0.2(0,0.3)	0.50(-5.71,7.12)
	Asbestos	0.77(0.49-1.19)	1.32(0.83-1.96)	71.64(17.1-145.68)	1.8(1.5,2.2)*	**2.39(0.99,3.81)** *
	Diesel engine exhaust	0.33(0.26-0.4)	0.42(0.34-0.52)	28.15(-1.29-65.92)	0.9(0.7,1.1)*	1.27(-5.88,8.98)
	Nickel	0.18(0.04-0.39)	0.19(0.04-0.43)	7.24(-16.19-36.07)	0.2(0.0,0.4)*	0.59(-5.73,7.35)
	Arsenic	0.16(0.06-0.26)	0.19(0.07-0.31)	17.03(-8.63-48.76)	0.5(0.3,0.8)*	0.90(-5.41,7.63)
	Polycyclic aromatic hydrocarbons	0.1(0.08-0.12)	0.13(0.1-0.16)	23.29(-3.74-57.46)	0.7(0.5,1.0)*	1.10(-5.42,8.07)
	Chromium	0.03(0.02-0.04)	0.04(0.03-0.04)	24.43(-3.76-58.13)	0.8(0.5,1.0)*	1.13(-8.62,11.92)
	Cadmium	0.02(0.01-0.02)	0.02(0.01-0.02)	17.18(-9.01-49.77)	0.6(0.3,0.8)*	0.91(-12.11,15.88)
	Beryllium	0.01(0.01-0.01)	0.01(0.01-0.01)	10.07(-13.98-39.26)	0.3(0.1,0.5)*	0.69(-18.78,24.82)
ASDR	Silica	27.02(12.56-43.21)	25.85(11.47-42.46)	-4.34(-26.62-22.79)	-0.2(-0.4,0.1)	0.49(-14.38,17.94)
	Asbestos	13.31(8.21-20.74)	20.89(12.78-31.62)	56.93(3.1-138.55)	1.5(1.2,1.7)*	2.42(1.56,3.29) *
	DE	9.97(8.02-12.15)	11.68(9.4-14.5)	17.23(-10.12-52.7)	0.6(0.3,0.8)*	1.27(-15.70,21.65)
	Nickel	5.34(1.1-11.75)	5.25(1.07-11.99)	-1.71(-23.79-25.31)	-0.1(-0.3,0.1)	0.59(-14.53,18.38)
	Arsenic	4.79(1.7-7.88)	5.15(1.85-8.62)	7.48(-16.54-37.45)	0.3(0.1,0.5)*	0.89(-14.15,18.57)
	PAHs	3.08(2.48-3.77)	3.49(2.73-4.35)	13.2(-11.24-45.86)	0.4(0.2,0.6)*	1.09(-14.39,19.37)
	Chromium	0.89(0.73-1.07)	1.01(0.82-1.25)	14.35(-11.69-46.42)	0.5(0.3-0.7)*	1.12(-14.46,19.55)
	Cadmium	0.47(0.37-0.58)	0.51(0.39-0.63)	7.58(-17.03-38.28)	0.3(0.1,0.5)*	0.91(-14.33,18.85)
	Beryllium	0.21(0.16-0.26)	0.21(0.17-0.27)	0.58(-21.86-27.79)	0(-0.2,0.2)	0.68(-14.82,19.01)

*Average annual percentage change or Netdrift was significantly different from 0 at the alpha=0.05 level

Table S4 Age-specific annual percentage change(Local drift) of occupational carcinogen attributable lung cancer ASMR in China from 1990 to 2019

Age	Silica	Asbestos	Diesel engine exhaust	Nickel	Arsenic	Polycyclic aromatic hydrocarbons	Chromium	Cadmium	Beryllium
27.5	-1.94(-3.71,-0.14)*	-3.55(-31.5,35.8)	-1.17(-2.96,0.65)	-1.82(-3.63,0.03)	-1.47(-3.27,0.36)	-1.25(-3.05,0.59)	-1.19(-3.91,1.60)	-1.48(-5.28,2.47)	-1.94(-7.67,4.15)
32.5	-1.94(-2.95,-0.92)*	0.9(-8.36,11.11)	-1.19(-2.21,-0.15)*	-1.80(-2.83,-0.75)*	-1.44(-2.47,-0.41)*	-1.24(-2.26,-0.20)*	-1.19(-2.74,0.39)	-1.45(-3.62,0.77)	-1.86(-5.16,1.56)
37.5	-2.36(-3.02,-1.69)*	1.89(-2.03,5.96)	-1.65(-2.32,-0.97)*	-2.23(-2.91,-1.55)*	-1.89(-2.56,-1.22)*	-1.71(-2.38,-1.03)*	-1.66(-2.68,-0.63)*	-1.90(-3.32,-0.45)*	-2.23(-4.39,-0.02)
42.5	-2.14(-2.53,-1.74)*	2.07(0.53,3.62)*	-1.42(-1.82,-1.02)*	-2.02(-2.43,-1.62)*	-1.70(-2.10,-1.30)*	-1.52(-1.92,-1.11)*	-1.48(-2.09,-0.86)*	-1.70(-2.55,-0.84)*	-1.97(-3.25,-0.66)
47.5	-1.33(-1.59,-1.06)*	2.91(2.14,3.68)*	-0.59(-0.87,-0.31)*	-1.22(-1.49,-0.94)*	-0.90(-1.17,-0.62)*	-0.71(-0.99,-0.43)*	-0.67(-1.09,-0.25)*	-0.89(-1.48,-0.30)*	-1.15(-2.03,-0.26)
52.5	-0.83(-1.05,-0.62)*	3.12(2.64,3.61)*	-0.1(-0.32,0.12)	-0.72(-0.94,-0.50)*	-0.39(-0.61,-0.17)*	-0.2(-0.42,0.02)	-0.16(-0.50,0.18)	-0.39(-0.86,0.08)	-0.69(-1.39,0.02)
57.5	-0.75(-0.94,-0.56)*	2.45(2.08,2.81)*	-0.01(-0.21,0.19)	-0.67(-0.86,-0.47)*	-0.34(-0.54,-0.14)*	-0.15(-0.35,0.06)	-0.11(-0.41,0.20)	-0.34(-0.76,0.08)	-0.62(-1.26,0.02)
62.5	0.04(-0.13,0.22)	2.06(1.76,2.36)*	0.80(0.62,0.98)*	0.12(-0.05,0.30)	0.42(0.24,0.60)*	0.61(0.43,0.80)*	0.65(0.37,0.92)*	0.44(0.05,0.82)*	0.25(-0.33,0.83)
67.5	0.49(0.29,0.68)*	1.82(1.56,2.08)*	1.26(1.05,1.46)*	0.55(0.34,0.75)*	0.82(0.62,1.03)*	1.02(0.81,1.23)*	1.04(0.73,1.36)*	0.85(0.41,1.29)*	0.7(0.04,1.37)
72.5	1.18(0.90,1.45)*	2.17(1.93,2.4)*	1.97(1.67,2.26)*	1.23(0.94,1.51)*	1.49(1.21,1.78)*	1.69(1.40,1.99)*	1.72(1.27,2.16)*	1.52(0.91,2.14)*	1.39(0.47,2.33)
77.5	2.33(1.84,2.82)*	3.13(2.90,3.36)*	3.16(2.63,3.69)*	2.39(1.88,2.90)*	2.65(2.13,3.16)*	2.85(2.33,3.38)*	2.87(2.08,3.68)*	2.67(1.58,3.78)*	2.55(0.9,4.23)
82.5	3.50(2.30,4.72)*	3.84(3.54,4.13)*	4.34(3.02,5.68)*	3.58(2.33,4.85)*	3.86(2.59,5.14)*	4.07(2.76,5.39)*	4.09(2.12,6.10)*	3.88(1.19,6.65)*	3.72(-0.35,7.96)
87.5	4.11(0.25,8.12)*	4.17(3.71,4.64)*	4.96(0.66,9.44)*	4.22(0.21,8.39)*	4.5(0.45,8.71)*	4.70(0.54,9.03)*	4.72(-1.52,11.36)	4.52(-3.94,13.72)	4.36(-8.27,18.74)
92.5	4.71(-16.41,31.16)	3.68(2.60,4.77)*	5.54(-18.15,36.09)	4.90(-16.78,32.23)	5.2(-16.57,32.65)	5.39(-16.99,33.81)	5.41(-26.65,51.49)	5.23(-35.91,72.79)	5.08(-51.01,125.43)

* Age-specific annual percentage change(Local drift) was significantly different from 0 at the alpha=0.05 level

Table S5 Age-specific annual percentage change(Local drift) of occupational carcinogen attributable lung cancer ASDR in China from 1990 to 2019

Age	Silica	Asbestos	Diesel engine exhaust	Nickel	Arsenic	Polycyclic aromatic hydrocarbons	Chromium	Cadmium	Beryllium
27.5	-1.93(-3.27,-0.58) *	-3.55(-20.99,17.74)	-1.16(-2.51,0.21)	-1.80(-3.18,-0.41)	-1.45(-2.81,-0.08) *	-1.23(-2.59,0.15)	-1.18(-2.53,0.20)	-1.47(-2.84,-0.08) *	-1.93(-3.30,-0.53) *
32.5	-1.93(-2.72,-1.13) *	0.89(-4.84,6.97)	-1.17(-1.97,-0.36) *	-1.78(-2.59,-0.96) *	-1.42(-2.22,-0.62) *	-1.22(-2.02,-0.41) *	-1.17(-1.97,-0.36) *	-1.43(-2.24,-0.62) *	-1.84(-2.65,-1.02) *
37.5	-2.34(-2.88,-1.80) *	1.92(-0.59,4.49)	-1.63(-2.17,-1.08) *	-2.21(-2.76,-1.66) *	-1.87(-2.42,-1.32) *	-1.68(-2.23,-1.13) *	-1.64(-2.18,-1.09) *	-1.88(-2.43,-1.32) *	-2.21(-2.76,-1.66) *
42.5	-2.11(-2.45,-1.77) *	2.1(1.07,3.15) *	-1.4(-1.74,-1.05) *	-2.00(-2.34,-1.65) *	-1.67(-2.02,-1.32) *	-1.49(-1.83,-1.14) *	-1.45(-1.79,-1.10) *	-1.67(-2.02,-1.32) *	-1.94(-2.29,-1.60) *
47.5	-1.30(-1.55,-1.05) *	2.95(2.39,3.51) *	-0.56(-0.81,-0.31) *	-1.19(-1.44,-0.94) *	-0.87(-1.12,-0.61) *	-0.68(-0.93,-0.42) *	-0.64(-0.89,-0.38) *	-0.86(-1.12,-0.61) *	-1.12(-1.37,-0.87) *
52.5	-0.80(-1.00,-0.59) *	3.2(2.82,3.58) *	-0.06(-0.27,0.16)	-0.68(-0.90,-0.47) *	-0.35(-0.57,-0.14) *	-0.16(-0.37,0.06) *	-0.12(-0.33,0.10)	-0.35(-0.56,-0.13) *	-0.65(-0.86,-0.43) *
57.5	-0.73(-0.93,-0.53) *	2.53(2.22,2.84) *	0.01(-0.19,0.22)	-0.65(-0.85,-0.44) *	-0.32(-0.53,-0.12) *	-0.13(-0.33,0.08)	-0.09(-0.29,0.12)	-0.32(-0.53,-0.11) *	-0.60(-0.80,-0.40) *
62.5	0.05(-0.14,0.25)	2.13(1.86,2.40) *	0.81(0.60,1.01) *	0.13(-0.07,0.34)	0.43(0.23,0.64) *	0.62(0.42,0.83) *	0.66(0.45,0.86) *	0.45(0.24,0.65) *	0.25(0.05,0.46) *
67.5	0.48(0.23,0.72) *	1.86(1.61,2.11) *	1.24(0.98,1.50) *	0.53(0.28,0.79) *	0.81(0.56,1.07) *	1.00(0.74,1.27) *	1.03(0.77,1.29) *	0.83(0.57,1.09) *	0.69(0.43,0.95) *
72.5	1.13(0.76,1.51) *	2.15(1.90,2.40)	1.92(1.52,2.33) *	1.18(0.79,1.57) *	1.45(1.05,1.84) *	1.65(1.25,2.05) *	1.67(1.27,2.07) *	1.48(1.08,1.88) *	1.35(0.95,1.75) *
77.5	2.29(1.54,3.03) *	3.07(2.79,3.35) *	3.11(2.31,3.93) *	2.34(1.56,3.11) *	2.60(1.81,3.38) *	2.80(2.01,3.60) *	2.82(2.02,3.63) *	2.62(1.84,3.42) *	2.50(1.72,3.29) *
82.5	3.45(1.38,5.57) *	3.82(3.41,4.23) *	4.29(2.02,6.61) *	3.53(1.37,5.73) *	3.80(1.63,6.03) *	4.01(1.78,6.29) *	4.04(1.8,6.32) *	3.83(1.63,6.08) *	3.67(1.47,5.92) *
87.5	4.06(-3.37,12.06)	4.21(3.49,4.94) *	4.91(-3.32,13.84)	4.16(-3.54,12.47)	4.44(-3.31,12.81)	4.64(-3.31,13.24)	4.67(-3.31,13.3)	4.46(-3.38,12.94)	4.31(-3.56,12.82)
92.5	4.64(-36.54,72.55)	3.75(1.88,5.65) *	5.47(-39.89,85.07)	4.83(-37.28,75.23)	5.13(-37.05,75.59)	5.32(-37.81,78.37)	5.34(-38,78.97)	5.16(-37.45,76.82)	5.02(-37.92,77.64)

* Age-specific annual percentage change(Local drift) was significantly different from 0 at the alpha=0.05 level

Table S6 Period effect of occupational carcinogen attributable lung cancer ASDR and ASMR in China from 1990 to 2019

Measure	Year	Silica	Asbestos	Diesel engine exhaust	Nickel	Arsenic	Polycyclic aromatic hydrocarbons	Chromium	Cadmium	Beryllium
ASMR	1992.5	0.9(0.48,1.71)	0.89(0.76,1.03)	0.85(0.41,1.78)	0.89(0.47,1.71)	0.87(0.46,1.67)	0.86(0.44,1.68)	0.86(0.31,2.36)	0.87(0.22,3.48)	0.89(0.10,7.68)
	1997.5	0.92(0.67,1.26)	0.83(0.76,0.91)*	0.89(0.61,1.28)	0.91(0.66,1.27)	0.9(0.65,1.25)	0.9(0.64,1.25)	0.89(0.54,1.49)	0.9(0.45,1.81)	0.91(0.31,2.7)
	2002.5	1	1	1	1	1	1	1	1	1
	2007.5	1(0.73,1.39)	1.29(1.19,1.40)*	1.05(0.73,1.52)	1.01(0.73,1.40)	1.03(0.74,1.42)	1.04(0.74,1.45)	1.04(0.63,1.73)	1.03(0.51,2.06)	1.02(0.34,2.99)
	2012.5	1(0.53,1.89)	1.55(1.34,1.78)*	1.1(0.53,2.28)	1.01(0.53,1.93)	1.05(0.55,2.00)	1.07(0.55,2.09)	1.07(0.39,2.96)	1.05(0.26,4.19)	1.02(0.12,8.81)
	2017.5	1.02(0.39,2.66)	1.33(1.08,1.63)*	1.16(0.39,3.48)	1.04(0.39,2.75)	1.09(0.41,2.87)	1.12(0.41,3.06)	1.13(0.25,5.16)	1.09(0.14,8.68)	1.06(0.04,26.61)
ASDR	1992.5	0.89(0.18,4.43)	0.88(0.8,0.97)*	0.84(0.13,5.28)	0.88(0.17,4.51)	0.86(0.17,4.33)	0.85(0.16,4.48)	0.85(0.16,4.52)	0.86(0.17,4.43)	0.88(0.17,4.7)
	1997.5	0.92(0.41,2.05)	0.82(0.76,0.88)*	0.89(0.35,2.22)	0.92(0.41,2.07)	0.91(0.40,2.03)	0.9(0.39,2.06)	0.9(0.39,2.07)	0.9(0.40,2.05)	0.92(0.40,2.12)
	2002.5	1	1	1	1	1	1	1	1	1
	2007.5	1(0.45,2.23)	1.29(1.22,1.38)*	1.05(0.42,2.63)	1.01(0.44,2.27)	1.02(0.46,2.30)	1.03(0.45,2.38)	1.04(0.45,2.40)	1.02(0.45,2.32)	1.01(0.44,2.33)
	2012.5	0.99(0.2,4.91)	1.55(1.41,1.70)*	1.09(0.17,6.79)	1(0.20,5.10)	1.04(0.21,5.21)	1.06(0.20,5.59)	1.06(0.20,5.68)	1.04(0.20,5.34)	1.01(0.19,5.4)
	2017.5	1.01(0.09,11.19)	1.32(1.16,1.50)*	1.15(0.07,18)	1.03(0.09,11.83)	1.08(0.10,12.17)	1.12(0.09,13.50)	1.12(0.09,13.79)	1.08(0.09,12.6)	1.05(0.09,12.92)

*Period RR was significantly different from 1 at the alpha=0.05 level.

Table S7 Birth cohort effect of occupational carcinogen attributable lung cancer ASMR in China from 1990 to 2019

Birth year	Silica	Asbestos	DE	Nickel	Arsenic	PAHs	Chromium	Cadmium	Beryllium
1905	0.38(0.1,1.38)	0.33(0.28,0.38) *	0.28(0.07,1.2)	0.37(0.10,1.41)	0.34(0.09,1.3) *	0.31(0.08,1.26)	0.31(0.04,2.57)	0.33(0.02,6.02)	0.35(0,28.82)
1910	0.47(0.31,0.69)*	0.4(0.36,0.44) *	0.37(0.24,0.56) *	0.46(0.30,0.69) *	0.42(0.28,0.64) *	0.4(0.26,0.61) *	0.4(0.21,0.76) *	0.42(0.17,1.02)	0.43(0.11,1.68)
1915	0.57(0.49,0.67) *	0.47(0.44,0.50) *	0.47(0.40,0.56) *	0.57(0.48,0.67) *	0.53(0.45,0.63) *	0.51(0.43,0.60) *	0.5(0.39,0.65) *	0.53(0.37,0.75) *	0.54(0.32,0.93) *
1920	0.73(0.67,0.80) *	0.6(0.57,0.63) *	0.62(0.57,0.68) *	0.73(0.67,0.79) *	0.69(0.63,0.76) *	0.66(0.61,0.73) *	0.66(0.58,0.76) *	0.69(0.57,0.83) *	0.7(0.52,0.93) *
1925	0.89(0.84,0.94) *	0.76(0.73,0.80) *	0.79(0.74,0.84) *	0.89(0.83,0.94) *	0.86(0.80,0.91) *	0.83(0.78,0.88) *	0.83(0.75,0.91) *	0.85(0.75,0.97) *	0.86(0.7,1.04)
1930	1(0.96,1.05)	0.89(0.85,0.93) *	0.92(0.88,0.97) *	1(0.95,1.05)	0.98(0.93,1.03)	0.96(0.91,1.01)	0.96(0.89,1.03)	0.97(0.88,1.08)	0.97(0.83,1.14)
1935	1.07(1.03,1.12)	0.97(0.93,1.02)	1.03(0.99,1.07)	1.07(1.03,1.12) *	1.06(1.02,1.11) *	1.05(1.01,1.10) *	1.05(0.99,1.13)	1.06(0.97,1.16)	1.05(0.92,1.21)
1940	1	1	1	1	1	1	1	1	1
1945	1.01(0.97,1.06)	1.06(1.00,1.12) *	1.05(1.00,1.09) *	1.02(0.98,1.06)	1.04(1.00,1.08) *	1.05(1.01,1.09) *	1.05(0.99,1.12)	1.04(0.95,1.14)	1.02(0.89,1.16)
1950	1.06(1.02,1.1) *	1.28(1.2,1.37) *	1.14(1.09,1.19) *	1.07(1.03,1.12) *	1.11(1.07,1.16) *	1.13(1.09,1.18) *	1.14(1.07,1.21) *	1.11(1.02,1.21) *	1.08(0.94,1.23)
1955	1.02(0.98,1.07)	1.52(1.41,1.64)*	1.14(1.09,1.19) *	1.04(0.99,1.09)	1.09(1.05,1.14) *	1.13(1.08,1.18) *	1.13(1.06,1.21) *	1.09(1.00,1.2) *	1.04(0.9,1.21)
1960	0.81(0.76,0.85) *	1.7(1.54,1.87) *	0.93(0.88,0.99) *	0.82(0.78,0.87) *	0.88(0.83,0.93) *	0.92(0.87,0.97) *	0.93(0.85,1.01)	0.88(0.78,0.99) *	0.82(0.68,0.98) *
1965	0.86(0.81,0.92) *	2.14(1.87,2.45) *	1.03(0.97,1.10)	0.89(0.84,0.95) *	0.97(0.91,1.03)	1.01(0.95,1.08)	1.02(0.93,1.12)	0.97(0.85,1.11)	0.9(0.74,1.1)
1970	0.75(0.69,0.82) *	2.08(1.65,2.62) *	0.94(0.86,1.02)	0.78(0.72,0.85) *	0.86(0.79,0.94) *	0.91(0.84,0.99) *	0.92(0.81,1.05)	0.86(0.72,1.03)	0.79(0.6,1.04)
1975	0.59(0.52,0.67) *	2.07(1.27,3.39) *	0.76(0.67,0.86) *	0.61(0.54,0.70) *	0.69(0.61,0.79) *	0.74(0.65,0.84) *	0.75(0.62,0.91) *	0.69(0.53,0.91) *	0.62(0.41,0.94) *
1980	0.55(0.44,0.68) *	2.61(0.7,9.67)	0.73(0.59,0.92) *	0.58(0.46,0.72) *	0.66(0.53,0.83) *	0.72(0.58,0.9) *	0.73(0.52,1.02)	0.66(0.41,1.06)	0.57(0.28,1.18)
1985	0.56(0.4,0.77) *	2.07(0.08,51.26)	0.79(0.57,1.09)	0.59(0.42,0.83) *	0.69(0.5,0.96) *	0.76(0.55,1.06)	0.78(0.47,1.28)	0.69(0.34,1.39)	0.58(0.2,1.72)
1990	0.53(0.29,0.95) *	0.58(0,74335.27)	0.77(0.42,1.38)	0.56(0.31,1.02)	0.66(0.36,1.2)	0.73(0.40,1.33)	0.75(0.31,1.84)	0.66(0.19,2.35)	0.56(0.08,3.89)

*Birth cohort RR was significantly different from 1 at the alpha=0.05 level.

Table S8 Birth cohort effect of occupational carcinogen attributable lung cancer ASDR in China from 1990 to 2019Cohort-dalys

Birth cohort	Silica	Asbestos	DE	Nickel	Arsenic	PAHs	Chromium	Cadmium	Beryllium
1905	0.38(0.03,4.92)*	0.33(0.26,0.41)*	0.29(0.02,4.83)	0.37(0.03,5.26)	0.34(0.02,4.87)	0.32(0.02,4.86)	0.32(0.02,4.87)	0.34(0.02,4.98)	0.35(0.02,5.23)
1910	0.47(0.24,0.94)*	0.4(0.35,0.46)*	0.37(0.18,0.78)*	0.46(0.23,0.95)*	0.43(0.21,0.89)*	0.41(0.19,0.85)*	0.4(0.19,0.85)*	0.43(0.21,0.89)*	0.44(0.21,0.91)*
1915	0.58(0.46,0.74)*	0.47(0.44,0.52)*	0.47(0.37,0.61)*	0.57(0.45,0.74)*	0.54(0.42,0.69)*	0.51(0.4,0.66)*	0.51(0.39,0.66)*	0.54(0.41,0.69)*	0.55(0.43,0.71)*
1920	0.74(0.66,0.83)*	0.61(0.57,0.65)*	0.63(0.56,0.72)*	0.74(0.65,0.83)*	0.7(0.62,0.79)*	0.67(0.59,0.76)*	0.67(0.59,0.76)*	0.70(0.61,0.79)*	0.71(0.62,0.80)*
1925	0.89(0.83,0.96)*	0.77(0.73,0.81)*	0.79(0.73,0.86)*	0.89(0.82,0.96)*	0.86(0.8,0.93)*	0.84(0.77,0.91)*	0.84(0.77,0.90)*	0.86(0.79,0.93)*	0.86(0.80,0.93)*
1930	1(0.95,1.06)	0.89(0.85,0.94)*	0.93(0.87,0.98)*	1(0.94,1.06)	0.98(0.92,1.04)	0.96(0.9,1.02)	0.96(0.90,1.02)	0.98(0.92,1.04)	0.98(0.92,1.03)
1935	1.07(1.02,1.13)*	0.97(0.93,1.02)	1.03(0.98,1.09)	1.08(1.02,1.13)	1.07(1.01,1.12)*	1.06(1.00,1.11)*	1.06(1.00,1.11)*	1.06(1.01,1.12)*	1.06(1.00,1.11)*
1940	1	1	1	1	1	1	1	1	1
1945	1.01(0.97,1.06)	1.08(1.02,1.13)*	1.05(1.00,1.10)*	1.02(0.97,1.07)	1.04(0.99,1.09)	1.05(1.00,1.10)*	1.05(1.00,1.10)*	1.04(0.99,1.09)	1.02(0.97,1.07)
1950	1.06(1.02,1.11)*	1.31(1.23,1.38)*	1.14(1.09,1.2)*	1.08(1.03,1.13)	1.12(1.07,1.17)*	1.14(1.09,1.19)*	1.14(1.09,1.20)*	1.12(1.07,1.17)*	1.08(1.03,1.13)*
1955	1.02(0.98,1.07)	1.54(1.44,1.65)*	1.14(1.09,1.2)*	1.04(1.00,1.1)	1.1(1.05,1.15)*	1.13(1.08,1.19)*	1.14(1.09,1.20)*	1.1(1.05,1.15)*	1.05(1.00,1.10)*
1960	0.81(0.77,0.86)*	1.73(1.60,1.87)*	0.94(0.89,0.99)*	0.83(0.78,0.88)*	0.89(0.84,0.94)*	0.92(0.87,0.98)*	0.93(0.88,0.99)*	0.89(0.84,0.94)*	0.82(0.78,0.87)*
1965	0.87(0.82,0.92)*	2.19(1.97,2.43)*	1.05(0.98,1.11)	0.9(0.85,0.96)*	0.98(0.92,1.04)	1.02(0.96,1.09)	1.03(0.97,1.1)	0.98(0.92,1.04)	0.91(0.86,0.97)*
1970	0.76(0.70,0.82)*	2.13(1.81,2.51)*	0.94(0.87,1.02)	0.79(0.73,0.85)*	0.87(0.8,0.94)*	0.92(0.85,0.99)*	0.93(0.86,1.01)	0.87(0.8,0.94)*	0.8(0.74,0.86)*
1975	0.6(0.53,0.66)*	2.13(1.53,2.96)*	0.77(0.69,0.86)*	0.62(0.56,0.70)*	0.7(0.63,0.78)*	0.75(0.67,0.84)*	0.76(0.68,0.85)*	0.7(0.63,0.78)*	0.63(0.56,0.70)*
1980	0.55(0.46,0.66)*	2.66(1.16,6.11)*	0.74(0.62,0.89)*	0.58(0.49,0.70)*	0.67(0.56,0.80)*	0.72(0.61,0.87)*	0.74(0.62,0.88)*	0.67(0.56,0.80)*	0.58(0.48,0.69)*
1985	0.57(0.44,0.73)*	2.1(0.30,14.67)	0.8(0.62,1.03)	0.6(0.46,0.78)*	0.7(0.54,0.91)*	0.77(0.60,1.00)	0.79(0.61,1.02)	0.70(0.54,0.91)*	0.59(0.46,0.77)*
1990	0.53(0.34,0.83)*	0.6(0,560.5)	0.77(0.5,1.21)	0.57(0.36,0.89)*	0.67(0.43,1.05)	0.74(0.47,1.16)	0.76(0.49,1.18)	0.67(0.43,1.05)	0.56(0.36,0.88)*

*Birth cohort RR was significantly different from 1 at the alpha=0.05 level.

Table S9 Predicted age-specific rate of lung cancer death attributable to occupational carcinogens

Year	Age group																	
	0-14	15-19	20-24	25-29	30-34	35-39	40-44	45-49	50-54	55-59	60-64	65-69	70-74	75-79	80-84	85-89	90-94	95+
2020	$6.41*10^{-7}$	$6.08*10^{-7}$	$9.08*10^{-5}$	$5.24*10^{-2}$	0.12	0.29	0.80	1.58	4.35	6.96	10.20	15.58	21.51	28.32	34.39	56.10	41.11	27.47
2021	$6.97*10^{-7}$	$6.45*10^{-7}$	$9.25*10^{-5}$	$5.29*10^{-2}$	0.12	0.29	0.79	1.51	4.10	7.27	9.60	15.69	21.33	28.86	33.74	57.09	42.09	29.06
2022	$7.66*10^{-7}$	$6.90*10^{-7}$	$9.48*10^{-5}$	$5.36*10^{-2}$	0.12	0.29	0.79	1.46	3.89	7.35	9.44	15.47	21.14	29.62	33.26	57.55	42.65	30.43
2023	$8.50*10^{-7}$	$7.47*10^{-7}$	$9.79*10^{-5}$	$5.45*10^{-2}$	0.12	0.29	0.80	1.43	3.71	7.18	9.74	14.78	21.11	30.33	33.00	57.43	43.27	31.81
2024	$9.52*10^{-7}$	$8.16*10^{-7}$	$1.02*10^{-4}$	$5.57*10^{-2}$	0.12	0.30	0.80	1.41	3.55	6.85	10.37	13.74	21.26	30.86	32.97	56.76	44.24	33.29
2025	$1.08*10^{-6}$	$9.03*10^{-7}$	$1.07*10^{-4}$	$5.72*10^{-2}$	0.13	0.30	0.81	1.40	3.38	6.48	11.07	12.66	21.57	30.91	33.27	55.82	45.41	34.72
2026	$1.24*10^{-6}$	$1.01*10^{-6}$	$1.14*10^{-4}$	$5.91*10^{-2}$	0.13	0.30	0.82	1.39	3.24	6.13	11.60	11.94	21.78	30.74	34.00	54.93	46.35	35.65
2027	$1.43*10^{-6}$	$1.15*10^{-6}$	$1.22*10^{-4}$	$6.15*10^{-2}$	0.13	0.30	0.83	1.39	3.14	5.83	11.76	11.79	21.56	30.57	35.01	54.33	46.88	36.25
2028	$1.69*10^{-6}$	$1.33*10^{-6}$	$1.33*10^{-4}$	$6.44*10^{-2}$	0.13	0.31	0.84	1.41	3.08	5.58	11.53	12.21	20.68	30.65	35.99	54.12	46.97	36.92
2029	$2.02*10^{-6}$	$1.56*10^{-6}$	$1.46*10^{-4}$	$6.81*10^{-2}$	0.14	0.31	0.85	1.42	3.06	5.36	11.05	13.06	19.30	31.00	36.78	54.32	46.63	37.92
2030	$2.46*10^{-6}$	$1.86*10^{-6}$	$1.63*10^{-4}$	$7.26*10^{-2}$	0.14	0.31	0.86	1.44	3.05	5.13	10.50	14.02	17.88	31.62	37.03	55.08	46.09	39.12
2031	$3.05*10^{-6}$	$2.26*10^{-6}$	$1.84*10^{-4}$	$7.82*10^{-2}$	0.15	0.32	0.86	1.47	3.05	4.94	9.99	14.77	16.96	32.11	37.04	56.61	45.61	40.15
2032	$3.83*10^{-6}$	$2.81*10^{-6}$	$2.12*10^{-4}$	$8.52*10^{-2}$	0.15	0.33	0.88	1.49	3.08	4.82	9.57	15.07	16.84	31.98	37.07	58.66	45.39	40.87
2033	$4.91*10^{-6}$	$3.56*10^{-6}$	$2.47*10^{-4}$	$9.39*10^{-2}$	0.16	0.34	0.89	1.52	3.13	4.77	9.22	14.88	17.57	30.89	37.42	60.72	45.53	41.22
2034	$6.41*10^{-6}$	$4.61*10^{-6}$	$2.94*10^{-4}$	0.11	0.17	0.35	0.91	1.55	3.19	4.77	8.92	14.36	18.93	29.04	38.13	62.52	46.03	41.22
2035	$8.54*10^{-6}$	$6.12*10^{-6}$	$3.56*10^{-4}$	0.12	0.18	0.36	0.93	1.58	3.26	4.78	8.61	13.76	20.48	27.12	39.20	63.45	47.06	41.08
2036	$1.16*10^{-5}$	$8.32*10^{-6}$	$4.41*10^{-4}$	0.14	0.20	0.38	0.96	1.61	3.34	4.84	8.37	13.20	21.77	25.95	40.17	64.03	48.80	41.02
2037	$1.61*10^{-5}$	$1.16*10^{-5}$	$5.57*10^{-4}$	0.16	0.22	0.40	0.99	1.65	3.43	4.93	8.24	12.76	22.42	26.02	40.38	64.68	51.04	41.20
2038	$2.29*10^{-5}$	$1.66*10^{-5}$	$7.21*10^{-4}$	0.19	0.25	0.43	1.03	1.69	3.54	5.06	8.24	12.43	22.36	27.42	39.40	65.96	53.38	41.75
2039	$3.34*10^{-5}$	$2.44*10^{-5}$	$9.55*10^{-4}$	0.23	0.28	0.46	1.07	1.75	3.64	5.22	8.32	12.15	21.83	29.87	37.46	67.96	55.56	42.68
2040	$4.97*10^{-5}$	$3.68*10^{-5}$	$1.30*10^{-3}$	0.28	0.32	0.50	1.13	1.81	3.75	5.39	8.45	11.87	21.16	32.71	35.39	70.71	57.06	44.15
2041	$7.57*10^{-5}$	$5.73*10^{-5}$	$1.81*10^{-3}$	0.35	0.37	0.55	1.20	1.88	3.87	5.60	8.65	11.68	20.57	35.21	34.31	73.38	58.33	46.37
2042	$1.18*10^{-4}$	$9.13*10^{-5}$	$2.60*10^{-3}$	0.45	0.44	0.61	1.28	1.97	4.02	5.83	8.94	11.67	20.15	36.77	34.88	74.79	59.74	49.18
2043	$1.88*10^{-4}$	$1.49*10^{-4}$	$3.84*10^{-3}$	0.60	0.52	0.69	1.39	2.07	4.19	6.09	9.32	11.84	19.92	37.22	37.30	74.06	61.82	52.19
2044	$3.07*10^{-4}$	$2.50*10^{-4}$	$5.84*10^{-3}$	0.81	0.64	0.79	1.52	2.20	4.39	6.37	9.76	12.14	19.78	36.90	41.28	71.53	64.71	55.19

Table S10 Predicted age-specific rate of lung cancer DALYs attributable to occupational carcinogens

Year	Age group																	
	0-14	15-19	20-24	25-29	30-34	35-39	40-44	45-49	50-54	55-59	60-64	65-69	70-74	75-79	80-84	85-89	90-94	95+
2020	8.72E-06	1.38E-06	6.69E-04	3.14	6.72	14.77	37.34	68.57	165.51	225.12	286.63	366.33	414.27	432.53	402.23	501.84	289.97	148.06
2021	9.10E-06	1.42E-06	6.73E-04	3.12	6.72	14.86	36.84	65.33	157.15	233.93	271.04	366.17	411.94	439.71	394.59	508.94	297.22	156.92
2022	9.56E-06	1.48E-06	6.80E-04	3.11	6.74	14.96	36.68	62.67	149.52	236.59	266.74	359.19	409.12	449.51	388.57	511.88	301.95	164.64
2023	1.01E-05	1.55E-06	6.91E-04	3.11	6.77	15.06	36.82	60.74	142.82	232.63	273.89	342.86	408.40	458.88	385.19	510.10	306.59	172.15
2024	1.08E-05	1.64E-06	7.07E-04	3.11	6.78	15.11	37.10	59.40	136.52	224.16	289.02	319.83	410.09	465.87	384.82	504.08	312.67	179.91
2025	1.16E-05	1.75E-06	7.28E-04	3.13	6.78	15.13	37.39	58.38	130.18	214.00	305.78	296.69	413.50	467.58	388.18	495.93	319.56	187.29
2026	1.27E-05	1.88E-06	7.55E-04	3.16	6.77	15.18	37.73	57.78	124.39	203.79	318.70	281.39	414.56	466.33	395.79	487.96	325.04	192.55
2027	1.39E-05	2.05E-06	7.90E-04	3.22	6.77	15.28	38.12	57.73	119.75	194.57	323.45	277.89	408.08	464.76	406.04	482.19	328.07	196.30
2028	1.54E-05	2.26E-06	8.33E-04	3.29	6.78	15.40	38.51	58.18	116.53	186.60	319.32	286.49	391.10	465.81	416.17	479.93	328.25	200.11
2029	1.71E-05	2.52E-06	8.88E-04	3.38	6.83	15.50	38.82	58.89	114.48	179.19	309.12	303.71	366.50	469.90	424.46	481.67	325.86	205.02
2030	1.93E-05	2.85E-06	9.55E-04	3.50	6.90	15.57	39.08	59.65	113.10	171.75	296.63	322.99	341.75	476.26	428.22	488.39	322.26	210.63
2031	2.19E-05	3.28E-06	1.04E-03	3.66	7.01	15.63	39.44	60.55	112.58	165.06	284.11	338.58	326.00	480.25	429.55	500.86	318.91	215.48
2032	2.51E-05	3.82E-06	1.14E-03	3.86	7.17	15.72	39.96	61.57	113.20	159.92	273.00	345.84	324.01	475.77	430.84	517.11	317.16	218.88
2033	2.93E-05	4.52E-06	1.28E-03	4.10	7.38	15.87	40.54	62.63	114.89	156.71	263.65	343.82	336.38	459.17	434.85	533.74	317.89	220.54
2034	3.46E-05	5.45E-06	1.44E-03	4.41	7.65	16.09	41.11	63.63	117.19	155.14	255.14	335.39	359.34	433.60	442.04	548.56	321.49	220.62
2035	4.15E-05	6.69E-06	1.66E-03	4.79	7.99	16.40	41.62	64.59	119.70	154.57	246.60	324.55	385.38	407.73	451.80	558.08	328.73	220.02
2036	5.02E-05	8.37E-06	1.93E-03	5.26	8.42	16.81	42.17	65.79	122.61	155.27	239.17	313.71	407.69	392.49	459.76	564.95	340.21	219.73
2037	6.16E-05	1.07E-05	2.30E-03	5.85	8.96	17.36	42.83	67.30	125.92	157.68	234.03	304.44	420.57	393.99	460.01	572.29	354.75	220.70
2038	7.65E-05	1.40E-05	2.78E-03	6.61	9.63	18.05	43.68	69.02	129.49	161.75	231.81	297.18	422.63	413.45	448.76	583.86	370.12	223.60
2039	9.63E-05	1.86E-05	3.42E-03	7.56	10.46	18.92	44.79	70.79	133.10	166.92	232.17	290.95	417.10	446.84	428.72	600.45	384.84	228.78
2040	1.23E-04	2.54E-05	4.31E-03	8.80	11.50	20.01	46.22	72.57	136.83	172.67	234.26	284.80	408.75	485.31	408.27	621.52	396.50	236.90
2041	1.61E-04	3.55E-05	5.55E-03	10.41	12.81	21.38	48.03	74.52	141.29	179.31	238.58	280.03	400.55	520.50	398.44	641.21	406.92	248.56
2042	2.15E-04	5.09E-05	7.33E-03	12.54	14.46	23.07	50.30	76.80	146.69	186.91	245.90	278.12	394.54	545.01	405.96	651.17	418.39	263.07
2043	2.91E-04	7.47E-05	9.91E-03	15.40	16.56	25.18	53.14	79.58	152.85	195.34	256.35	279.96	391.39	556.58	432.93	645.56	433.79	278.93
2044	4.01E-04	1.12E-04	1.37E-02	19.30	19.28	27.82	56.68	83.01	159.51	204.37	269.21	285.36	389.95	559.01	476.16	627.64	454.00	295.14

Table S11 Contribution of changes in population aging, population growth, and age-specific death rate to the net change of lung cancer from occupational carcinogens in China from 1991 to 2044*.

Year	Population aging, n(%)	Age-specific rate ,n(%)	Population growth, n(%)	Net change, n (%)
1991	300(1.5)	185(0.9)	261(1.3)	746(3.6)
1992	330(1.6)	463(2.3)	515(2.5)	1308(6.4)
1993	952(4.6)	341(1.7)	754(3.7)	2047(10)
1994	1331(6.5)	340(1.7)	986(4.8)	2657(12.9)
1995	1745(8.5)	527(2.6)	1214(5.9)	3486(16.9)
1996	2230(10.8)	865(4.2)	1443(7)	4538(22.1)
1997	2740(13.3)	1049(5.1)	1664(8.1)	5453(26.5)
1998	3277(15.9)	1296(6.3)	1885(9.2)	6458(31.4)
1999	3927(19.1)	1840(8.9)	2117(10.3)	7884(38.3)
2000	4624(22.5)	2930(14.2)	2370(11.5)	9924(48.2)
2001	5408(26.3)	3701(18.0)	2611(12.7)	11720(57)
2002	6242(30.3)	4625(22.5)	2857(13.9)	13724(66.7)
2003	7137(34.7)	5446(26.5)	3097(15.1)	15680(76.2)
2004	8126(39.5)	6220(30.2)	3334(16.2)	17680(85.9)
2005	9105(44.3)	6560(31.9)	3543(17.2)	19208(93.4)
2006	10162(49.4)	6658(32.4)	3744(18.2)	20564(100)
2007	11324(55.0)	7163(34.8)	3979(19.3)	22466(109.2)
2008	12667(61.6)	8340(40.5)	4266(20.7)	25273(122.9)
2009	14087(68.5)	9186(44.7)	4541(22.1)	27814(135.2)
2010	15611(75.9)	10487(51.0)	4849(23.6)	30947(150.4)
2011	16960(82.4)	10726(52.1)	5087(24.7)	32773(159.3)
2012	18140(88.2)	10415(50.6)	5301(25.8)	33856(164.6)
2013	19088(92.8)	9295(45.2)	5464(26.6)	33847(164.5)
2014	20191(98.1)	8571(41.7)	5656(27.5)	34418(167.3)
2015	21343(103.7)	7998(38.9)	5885(28.6)	35226(171.2)
2016	22538(109.6)	7642(37.1)	6177(30)	36357(176.7)
2017	23758(115.5)	7440(36.2)	6478(31.5)	37676(183.1)
2018	25143(122.2)	7712(37.5)	6788(33)	39643(192.7)
2019	26780(130.2)	8388(40.8)	7120(34.6)	42288(205.6)
2020	25675(124.8)	9151(44.5)	7562(36.8)	42388(206)
2021	27214(132.3)	9265(45.0)	7842(38.1)	44321(215.4)
2022	28744(139.7)	9365(45.5)	8109(39.4)	46218(224.7)
2023	30253(147.1)	9400(45.7)	8355(40.6)	48008(233.4)
2024	31779(154.5)	9426(45.8)	8589(41.8)	49794(242)
2025	33331(162.0)	9460(46.0)	8813(42.8)	51604(250.8)
2026	35014(170.2)	9715(47.2)	9049(44)	53778(261.4)
2027	36646(178.1)	9946(48.3)	9268(45)	55860(271.5)
2028	38207(185.7)	10109(49.1)	9462(46)	57778(280.9)
2029	39745(193.2)	10266(49.9)	9640(46.9)	59651(290)
2030	41303(200.8)	10505(51.1)	9811(47.7)	61619(299.5)
2031	42960(208.8)	11034(53.6)	9997(48.6)	63991(311.1)

2032	44547(216.5)	11591(56.3)	10167(49.4)	66305(322.3)
2033	46074(224)	12145(59.0)	10319(50.2)	68538(333.2)
2034	47636(231.6)	12734(61.9)	10462(50.9)	70832(344.3)
2035	49313(239.7)	13455(65.4)	10610(51.6)	73378(356.7)
2036	51234(249)	14567(70.8)	10788(52.4)	76589(372.3)
2037	53219(258.7)	15843(77.0)	10970(53.3)	80032(389)
2038	55210(268.4)	17245(83.8)	11147(54.2)	83602(406.4)
2039	57202(278.1)	18765(91.2)	11316(55)	87283(424.3)
2040	59236(287.9)	20473(99.5)	11485(55.8)	91194(443.3)
2041	61564(299.3)	22716(110.4)	11693(56.8)	95973(466.5)
2042	63862(310.4)	25150(122.3)	11895(57.8)	100907(490.5)
2043	66084(321.2)	27752(134.9)	12086(58.7)	105922(514.9)
2044	68306 (332.0)	30612 (148.8)	12277 (59.7)	111195(540.5)

*Using 1990 as the reference year.

Table S12 Contribution of changes in population aging, population growth, and age-specific rate to the net change of lung cancer DALYs from occupational carcinogens in China from 1991 to 2044*.

Year	Population aging, n(%)	Age-specific rate ,n(%)	Population growth, n(%)	Net change, n (%)
1991	8084(1.4)	4075(0.7)	7435(1.3)	19593(3.3)
1992	14448(2.5)	2637(0.4)	14520(2.5)	31605(5.4)
1993	25591(4.4)	5810(1.0)	21415(3.6)	52816(9.0)
1994	36012(6.1)	5660(1.0)	27977(4.8)	69649(11.9)
1995	47351(8.1)	11141(1.9)	34448(5.9)	92939(15.8)
1996	60586(10.3)	18614(3.2)	40860(7.0)	120060(20.5)
1997	74291(12.7)	21907(3.7)	47034(8.0)	143231(24.4)
1998	88514(15.1)	27634(4.7)	53187(9.1)	169334(28.9)
1999	105790(18.0)	39877(6.8)	59578(10.2)	205245(35.0)
2000	123972(21.1)	65764(11.2)	66445(11.3)	256181(43.7)
2001	144321(24.6)	80218(13.7)	72794(12.4)	297332(50.7)
2002	165877(28.3)	99109(16.9)	79277(13.5)	344263(58.7)
2003	188754(32.2)	111483(19.0)	85333(14.5)	385569(65.7)
2004	214444(36.5)	125397(21.4)	91455(15.6)	431296(73.5)
2005	239767(40.9)	126903(21.6)	96713(16.5)	463383(79.0)
2006	266288(45.4)	122765(20.9)	101751(17.3)	490804(83.7)
2007	294211(50.1)	128636(21.9)	107543(18.3)	530391(90.4)
2008	323768(55.2)	144319(24.6)	114069(19.4)	582156(99.2)
2009	353845(60.3)	149455(25.5)	119996(20.5)	623297(106.2)
2010	387631(66.1)	169345(28.9)	126957(21.6)	683933(116.6)
2011	418758(71.4)	169655(28.9)	132618(22.6)	721031(122.9)
2012	446679(76.1)	159930(27.3)	137994(23.5)	744602(126.9)
2013	469434(80.0)	132074(22.5)	142233(24.2)	743741(126.8)
2014	494136(84.2)	111982(19.1)	146949(25.0)	753067(128.4)
2015	521338(88.9)	97416(16.6)	152751(26.0)	771505(131.5)
2016	547965(93.4)	87995(15.0)	160024(27.3)	795984(135.7)

2017	574176(97.9)	81886(14.0)	167420(28.5)	823482(140.4)
2018	604295(103.0)	88418(15.1)	175013(29.8)	867725(147.9)
2019	637889(108.7)	102534(17.5)	182720(31.1)	923143(157.3)
2020	610616(104.1)	86531(14.7)	191428(32.6)	888576(151.4)
2021	637419(108.6)	79400(13.5)	196810(33.5)	913628(155.7)
2022	664315(113.2)	72296(12.3)	201834(34.4)	938446(159.9)
2023	690633(117.7)	64147(10.9)	206370(35.2)	961150(163.8)
2024	718218(122.4)	57410(9.8)	210729(35.9)	986357(168.1)
2025	745522(127.1)	50538(8.6)	214712(36.6)	1010772(172.3)
2026	770748(131.4)	45674(7.8)	218276(37.2)	1034698(176.4)
2027	794991(135.5)	40947(7.0)	221434(37.7)	1057371(180.2)
2028	817952(139.4)	35865(6.1)	224122(38.2)	1077939(183.7)
2029	839988(143.2)	31085(5.3)	226410(38.6)	1097484(187.1)
2030	861383(146.8)	27258(4.6)	228371(38.9)	1117011(190.4)
2031	879416(149.9)	26669(4.5)	229895(39.2)	1135979(193.6)
2032	896462(152.8)	28149(4.8)	231205(39.4)	1155817(197.0)
2033	912876(155.6)	31100(5.3)	232296(39.6)	1176272(200.5)
2034	929951(158.5)	35432(6.0)	233261(39.8)	1198645(204.3)
2035	948510(161.7)	41158(7.0)	234163(39.9)	1223831(208.6)
2036	966464(164.7)	51252(8.7)	234974(40.0)	1252690(213.5)
2037	986428(168.1)	65283(11.1)	235993(40.2)	1287703(219.5)
2038	1007685(171.7)	82930(14.1)	237129(40.4)	1327743(226.3)
2039	1029725(175.5)	103687(17.7)	238278(40.6)	1371690(233.8)
2040	1052187(179.3)	127122(21.7)	239351(40.8)	1418660(241.8)
2041	1073781(183.0)	156696(26.7)	240421(41.0)	1470898(250.7)
2042	1096552(186.9)	192102(32.7)	241728(41.2)	1530382(260.8)
2043	1120349(191.0)	233630(39.8)	243266(41.5)	1597244(272.2)
2044	1145451(195.2)	281623(48.0)	245037(41.8)	1672111(285.0)

*Using 1990 as the reference year.
