# Supplementary figures and images for: Burden of Lung Cancer Attributable to Occupational Carcinogens from 1990 to 2019 and Projections until 2044 in China
Source: Cancers (Basel). 2022 Aug 11;14(16):3883. doi: 10.3390/cancers14163883 (PMC9405822; doi:10.3390/cancers14163883)

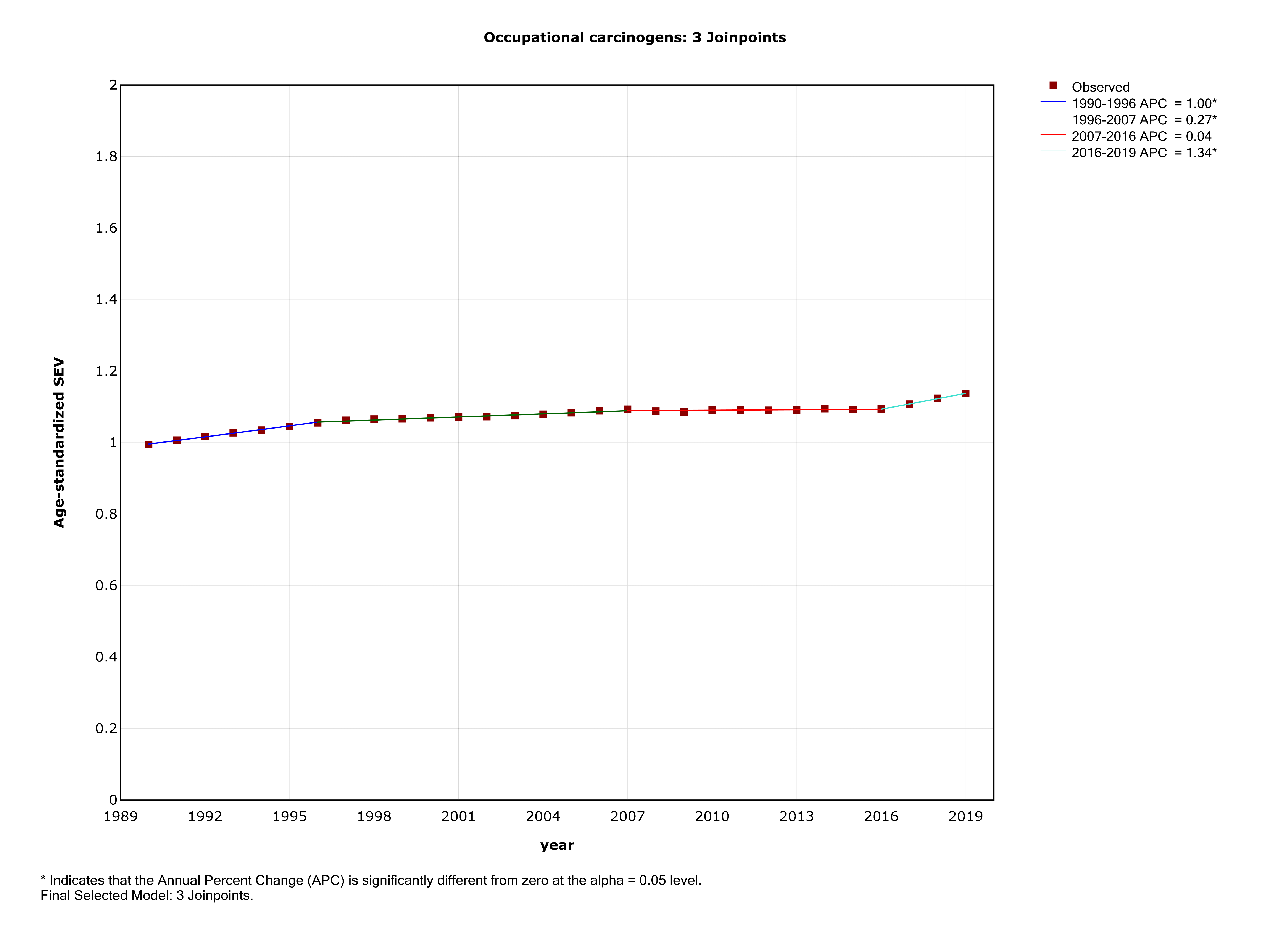

Supplement: Supplementary file 1 [file cancers-14-03883-s001.zip › cancers-1775604-supplementary/cancers-1775604-supplementary fig1.tif]
